# Major complications of percutaneous native and transplant kidney biopsy: a complete 10-year national prospective cohort study

**DOI:** 10.1093/ckj/sfaf196

**Published:** 2025-06-23

**Authors:** Colin C Geddes, Samira Bell, Kate Buck, Bryan Conway, Vishal Dey, Robert Hunter, Nicola Joss, Michael Kelly, Joe Lakey, Steve Marjoribanks, Wendy Metcalfe, Shona Methven, Lisa Norman, Kate Stevens, Graham Stewart, Jamie Traynor, David Walbaum, Wan Wong, Emily McQuarrie

**Affiliations:** Glasgow Renal and Transplant Unit, Queen Elizabeth University Hospital Glasgow, UK; Renal Unit, Ninewells Hospital Dundee; Renal Unit, Victoria Hospital Kirkcaldy; Renal Unit, Royal Infirmary of Edinburgh; Renal Unit, University Hospital Crosshouse; Renal Unit, Royal Infirmary of Edinburgh; Renal Unit, Raigmore Hospital Inverness; Renal Unit, Dumfries and Galloway Royal Infirmary; Public Health Scotland; Public Health Scotland; Renal Unit, Royal Infirmary of Edinburgh; Renal Unit, Aberdeen Royal Infirmary; Renal Unit, Royal Infirmary of Edinburgh; Glasgow Renal and Transplant Unit, Queen Elizabeth University Hospital Glasgow, UK; Renal Unit, Ninewells Hospital Dundee; Glasgow Renal and Transplant Unit, Queen Elizabeth University Hospital Glasgow, UK; Renal Unit, Aberdeen Royal Infirmary; Renal Unit, University Hospital Monklands, Airdrie; Glasgow Renal and Transplant Unit, Queen Elizabeth University Hospital Glasgow, UK

**Keywords:** acute kidney injury, complication, haemorrhage, kidney biopsy, kidney transplant

## Abstract

**Background:**

Previous reports of incidence of major complications (MC) of kidney biopsy vary depending on definitions of MC, single or multicentre analysis, and prospective or retrospective data collection. We aimed to provide accurate, unbiased information about the incidence of MC by analysing 10-year data from a prospective national renal biopsy registry.

**Methods:**

The Scottish Renal Biopsy Registry has prospectively collected data on all native and transplant kidney biopsies undertaken in the nine adult renal centres in Scotland since 2014. Nephrologists from each centre report demographics, operator, coded indication, coded diagnosis and coded MC.

**Results:**

A total of 8476 biopsies were reported in the 10 years between 2014 and 2023 (6167 native, 2309 transplant). The overall incidences of MC following native and transplant kidney biopsy were 2.1% and 1.4%, respectively (*P *< .001). The most common MC of native kidney biopsy was the requirement for ‘arteriography with embolization’ (0.63% of biopsies) and the most common MC of transplant biopsy was ‘blood transfusion only’ (0.30%). Nine deaths (0.15%) and no nephrectomies were attributed to native biopsy, and one death and one nephrectomy were attributed to transplant biopsy. MC were more common in women than men (2.2 vs 1.5%; *P *= .01). MC incidence was identical for biopsies performed by nephrologists (*n* = 5373) and radiologists (*n* = 2709). A positive association between quartile of serum creatinine at the time of native biopsy and incidence of MC diminished when acute kidney injury as indication for biopsy was excluded. Transplant biopsies >10 years after transplant had a higher risk of MC (3.4%).

**Conclusion:**

MC of kidney biopsy in the modern era remain rare. This registry analysis provides accurate estimates of risk based on unbiased national data. The increased incidence of MC in women merits further study.

KEY LEARNING POINTS **What was known:**

- Major complications (MC) of kidney biopsy are rare.
- Previous reports of the risk of MC of kidney biopsy were limited because of variation in definition of major complication, small numbers of patients included, bias related to single centre analysis, variations in biopsy practice or a combination of these.

**This study adds:**

- This is the first complete national analysis reporting pre-defined and verifiably attributed MC.
- The incidence was higher in native than transplant biopsies and in women than men.

**Potential impact:**

- Clinicians and patients can refer to these data when discussing the risks and benefits of kidney biopsy.
- Researchers can refer to these data when designing studies to reduce the risk of MC of kidney biopsy.

## INTRODUCTION

Kidney biopsy is the definitive pathological test for intrinsic kidney disease. The major risk of kidney biopsy is bleeding complications. The fact that the kidney is highly vascular and all successful kidney biopsies contain sheared blood vessels means that post-procedure bleeding is inevitable. However, clinically important bleeding complications are rare due to the effectiveness of normal physiological mechanisms to limit bleeding from traumatized blood vessels and clinical strategies to mitigate bleeding. It is difficult to be precise about the risk and how to minimize complications because published studies vary depending on definition of a major complication (MC), native or transplant biopsy, single or multicentre analysis, prospective or retrospective data collection [1, 2]. In the largest published meta-analysis of MC of native kidney biopsy, the median number of patients in the included studies was only 210 [1]. Furthermore, biopsy practice also varies worldwide in terms of patient preparation and patient observation (post-procedure imaging, haemoglobin check, duration of post-procedure observation).

The Scottish Renal Biopsy Registry has prospectively collected data on all native kidney biopsies undertaken in the nine adult renal centres in Scotland since 2014 and all transplant biopsies since 2015 [3]. Nephrologists from each centre report data on every biopsy performed in their centre including information on coded MC [4]. In this study we report the incidence of pre-defined MC of native and transplant kidney biopsies in a complete national dataset spanning 10 years, and explore underlying associations.

## MATERIALS AND METHODS

All native and transplant kidney biopsies reported to the Scottish Renal Biopsy Registry in the 10 years between 2014 and 2023 were included. This registry covers all nine adult renal centres in the National Health Service of Scotland that serves the population of approximately 5 million. Scotland is part of the UK but the health service in Scotland is devolved, meaning that it exists independently from the health service in the rest of the UK. It is free to patients at the point of access. No diagnostic kidney biopsies are conducted in the much smaller private health sector. Data on age, sex, kidney function at the time of biopsy (serum creatinine), operator (nephrologist or radiologist, accredited nephrologist or trainee nephrologist), biopsy indication, biopsy adequacy, biopsy diagnosis and MC are reported by the nephrology representative for each centre using a pre-defined electronic template. Biopsy indication is recorded using coded terms defined by the biopsy registry steering group. Native biopsy main diagnosis within the registry is defined as the European Renal Association primary renal disease coded term [5] that best explained the indication for that biopsy with the addition of ‘no kidney tissue obtained’, ‘kidney biopsy result normal’, ‘other’ and ‘thrombotic microangiopathy’. Transplant kidney biopsy diagnosis is recorded using coded terms defined by the biopsy registry steering group based on the Banff classification for kidney allograft pathology [6]. In the case of kidney transplant biopsies, the time since latest kidney transplant was derived through data linkage within the Scottish Renal Biopsy Registry.

MC of kidney biopsy are recorded prospectively by each centre using coded terms defined by the steering group for events that are judged by the reporting nephrologist to be directly attributable to the biopsy: ‘arteriography with embolization’, ‘arteriography without embolization’, ‘clot retention with no cystoscopic intervention’, ‘clot retention with cystoscopic intervention’, ‘blood transfusion only’, ‘death within one month directly attributable to biopsy’ and ‘nephrectomy’. For each instance of MC following biopsy, the most serious was reported. For example, a biopsy associated with blood transfusion and arteriography with embolization is recorded as ‘arteriography with embolization’. This means that the total number of MC is also the total number of biopsies that caused a MC.

All centres perform native kidney biopsy and seven of the nine centres perform transplant kidney biopsy. The majority of transplant biopsies are performed at the two Scottish kidney transplanting centres. In all centres, kidney biopsies are performed under direct ultrasound guidance by nephrologists or radiologists using 16 G or 18 G spring loaded biopsy guns. Most nephrologists who perform kidney biopsy in Scotland are trained initially on a cadaver simulation course [7]. All centres discontinue clopidogrel, direct oral anticoagulants and warfarin prior to the procedure. Three centres discontinue aspirin for 7 days before non-urgent biopsy. Pre-procedure optimization includes ensuring blood pressure is controlled and blood tests indicate adequate clotting factors. Three of the centres use DDAVP (desmopressin) prior to the procedure in occasional cases felt to be at particular risk of bleeding complications. The other six centres never use DDAVP. No centres perform post-biopsy imaging as routine and all centres have a 6-h post-procedure bed rest and observation protocol. All centres have on-site access to interventional radiologists for bleeding complications although the three smallest centres do not have this 24 h per day 7 days per week.

### Analysis and statistics

The subjects of the analysis were biopsy procedures. As expected some patients had more than one biopsy procedure during the 10-year period. We analysed the incidence of each MC category as a percentage of all kidney biopsies. Sub-analyses of the association between incidence of MC and age, sex, operator, centre volume, centre that continues aspirin for elective biopsies, indication for biopsy, serum creatinine at the time of biopsy, biopsy diagnosis and time since kidney transplant were then conducted. Age and serum creatinine at the time of biopsy were reported as continuous variables but converted to decades of age and quartiles of serum creatinine for analysis. Centre volume was analysed for native biopsies by tertile of total number of biopsies (i.e. comparing the MC incidence in three groups of three centres ranked by the number of reported biopsies). As only seven centres perform transplant biopsies, we compared the three centres with the largest and the four centres with the smallest number of transplants. For comparisons of the incidence of MC in these sub-analyses we performed chi-square tests. Statistical tests were performed in Microsoft Excel. A *P*-value <.05 was regarded as statistically significant.

### Ethics and approval

The Scottish Renal Registry sits within the Scottish National Audit Programme which is part of Public Health Scotland and governed by the Scottish Government National Audit Programme Board. The steering group has representation from all nine centres. Formal ethical approval was waived according to Public Health Scotland Information Governance as analysis of routinely collected data and individual patient consent was not required. Access and use of the data for the purpose of this work were approved following a Public Health Scotland information governance review of linking internal datasets. No external funding was required.

## RESULTS

In total, 8476 biopsies were reported in the 10 years between 2014 and 2023 (6167 native biopsies in 5670 patients, 2309 transplant biopsies in 1386 patients). Table 1 describes the demographics and operator by biopsy type. In approximately one-third of cases, biopsies were undertaken by radiologists and in two-thirds of cases, biopsies were undertaken by nephrologists (senior trainees or accredited nephrologist) (Table 1).

**Table 1:** Clinical characteristics of native and transplant kidney biopsies reported between 2014 and 2023.

	Native	Transplant
Total number	6167	2309
% men	55.1	58.6
Mean age in years (SD)	57.1 (17.3)	48.0 (14.4)
Operator, *n* (%)		
Radiologist	1860 (30.2)	849 (36.8)
Nephrologist	3956 (64.1)	1417 (61.4)
Not stated	351 (5.7)	43 (1.7)
Median days since last transplant (interquartile range)	NA	163 (22–1169)

NA, not applicable.

### Patient characteristics

Both native and transplant kidney biopsy were more frequent in men than women. The mean age at the time of transplant biopsy was lower than for native (48.0 vs 57.1 years). The median time from last kidney transplant to kidney transplant biopsy was 163 days (interquartile range 22–1169) (Table 1).

### Major complications

A total of 155 MC were reported with overall incidences of MC following native and transplant kidney biopsy of 2.1% and 1.4%, respectively (*P *< .001) (Table 2). The most common MC of native kidney biopsy was ‘arteriography with embolization’ (0.63% of biopsies) and the most common MC of transplant biopsy was ‘blood transfusion only’ (0.30%). There were nine deaths within 28 days attributable to native kidney biopsy (0.15%) and one death attributable to transplant biopsy (0.04%). There were 22 MC in the ‘other’ category. These comprised cases that were felt by the reporting nephrologists to constitute MC that did not fit into the pre-defined categories above: spleen biopsy, liver biopsy, bowel biopsy, prolonged frank haematuria, prolonged pain, readmission to hospital for investigation of pain. The incidence of MC was similar in first and in subsequent biopsies so subsequent sub-analyses included the whole cohort.

**Table 2:** Reported MC in native and transplant biopsies.

	Native	Transplant
	(*n* = 6167)	(*n* = 2309)
Arteriography and embolization	39 (0.63)	3 (0.13)
Arteriography no embolization	20 (0.32)	2 (0.09)
Blood transfusion only	21 (0.34)	7 (0.30)
Clot obstruction with no cystoscopic intervention	12 (0.19)	5 (0.22)
Clot obstruction with cystoscopic intervention	5 (0.08)	4 (0.17)
Other	21 (0.34)	1 (0.04)
Surgery no nephrectomy	0	4 (0.17)
Nephrectomy	0	1 (0.04)
Death	9 (0.15)	1 (0.04)
Total	127 (2.1)	28 (1.2)

Data are presented as *n* (%).

### Age

**Figure 1:**
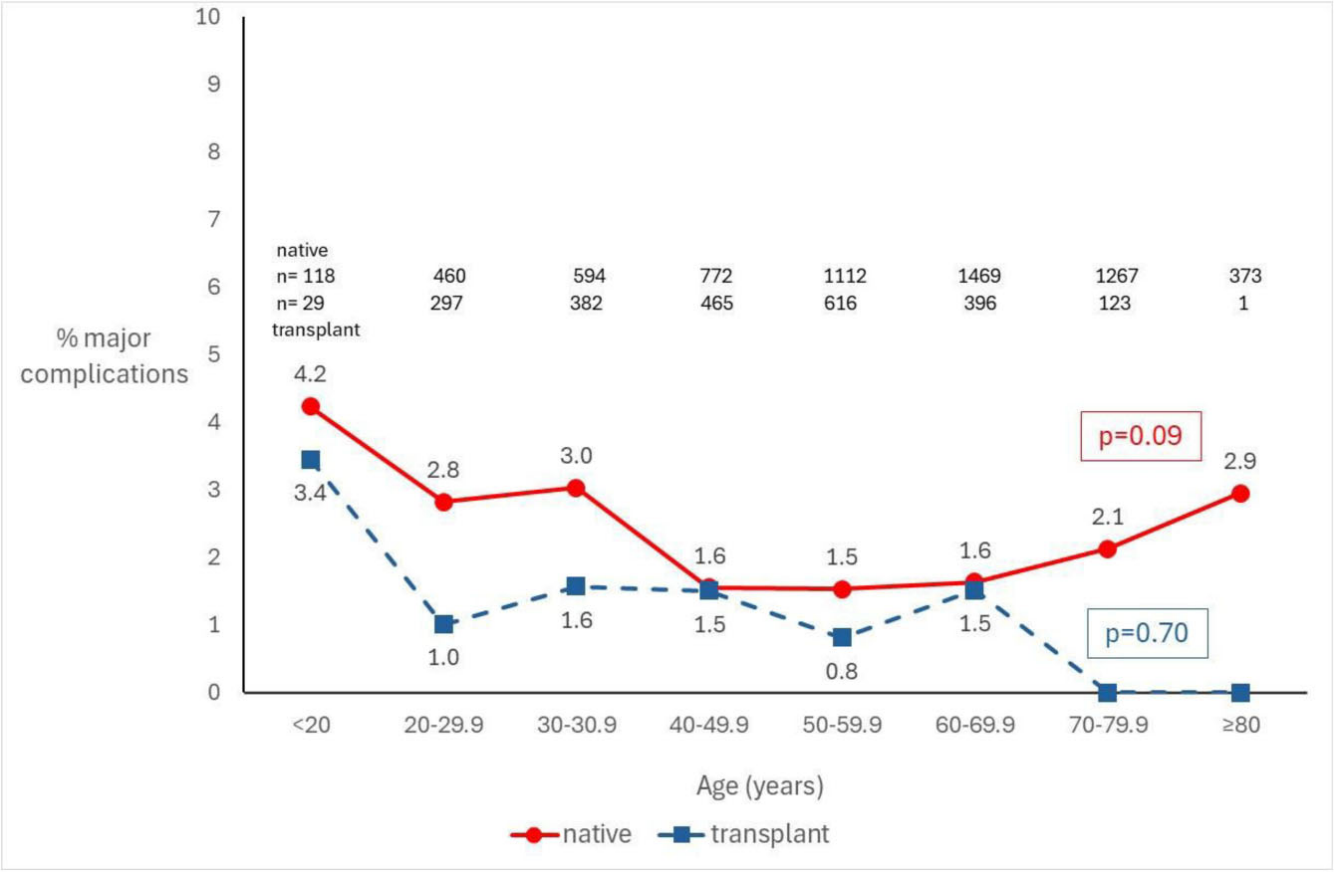
Incidence of MC of native and transplant kidney biopsy by age category.

The age distribution and incidence of MC is shown in Fig. 1. Patients in the <20 years age category had the highest incidence of MC of native biopsy (4.2%) and there was the impression of a U-shaped relationship between age and incidence of major complications that was not statistically significant. There was no obvious association between age category and incidence of MC of transplant biopsy.

### Sex

The sex distribution of native and transplant biopsy is shown in Table 3. The incidence of MC was numerically higher in women than men for both native biopsy (2.4 vs 1.7%; *P *= .06) and transplant biopsy (1.6 vs 1.0%; *P *= .19). When native and transplant kidney biopsies are combined the incidence of MC was 2.2% in women and 1.5% in men (*P *= .01).

**Table 3:** MC of native and transplant kidney biopsy by sex, operator, centre volume and whether or not the biopsy was performed in a centre that stops aspirin for 7 days for an elective biopsy.

	Native (*n* = 6167)				Transplant (*n* = 2309)			
	No. of biopsies	No. of MC	%	*P*	No. of biopsies	No. of MC	%	*P*
Sex								
Female	2715	66	2.4	.06	956	15	1.6	.19
Male	3400	59	1.7		1353	13	1.0	
Not stated	52	2	3.8		0	NA	NA	
Operator								
Nephrologist—all	3956	85	2.1	.9	1417	17	1.2	.96
Radiologist	1860	39	2.1		849	10	1.2	
Nephrologist—accredited^a^	1408	26	1.8	.39	337	2	0.6	.21
Nephrologist—trainee^a^	2520	57	2.3		1031	15	1.5	
Not stated	351	3	0.9		43	1	2.3	
Centre volume								
Tertile 1 (*n* = 752–1848)	4007	82	2.0	.99				
Tertile 2 (*n* = 429–524)	1448	30	2.1					
Tertile 3 (150–297)	712	15	2.1					
Aspirin								
Centre continues aspirin	4066	85	2.1	.81	1334	17	1.3	.75
Centre stops aspirin	2101	42	2.0		975	11	1.1	

a In 28 native biopsies and 49 transplant biopsies performed by nephrologists the seniority of the nephrologist was not stated.

NA, not applicable.

### Operator

The distribution and incidence of MC by operator is shown in Table 3. The incidence of MC was the same for biopsies performed by radiologists and nephrologists both for native (2.1 vs 2.1%; *P *= .9) and transplant (1.2 vs 1.2%; *P *= .96) biopsies. In biopsies performed by nephrologists we were also able to compare biopsies performed by accredited (senior) nephrologists and nephrologists in training. In both native and transplant biopsies the incidence of MC were not significantly lower in biopsies performed by senior nephrologists than biopsies performed by training nephrologists.

### Centre volume

The analysis of tertiles of centre volume for native biopsy is shown in Table 3. This produced three distinct groups for comparison on the basis of volume with the three largest centres performing between 752 and 1848 native biopsies during the study period, the three smallest performing between 150 and 297 biopsies and the intermediate group of three centres 429–524 biopsies. The incidence of MC was remarkably similar between the three groups at 2.0, 2.1 and 2.1%, respectively (*P *= .99). As only seven centres perform transplant biopsy, we assessed centre volume by comparing the three largest centres (228–990 biopsies) with the four smallest centres (51–99 biopsies). Again, the incidence of MC was similar at 1.2 vs 1.0% (*P *= .79).

### Centres that continue aspirin

We were unable to include medication information for individual patients, but we were able to analyse MC by whether the biopsy was performed in a centre that continues aspirin in all patients or a centre that routinely stops aspirin for 7 days before a non-urgent biopsy. There was no significant difference in MC in either native (2.1 vs 2.0%; *P *= .81) or transplant biopsies (1.3 vs 1.1%; *P *= .75) (Table 3).

### Indication for biopsy

The distribution and incidence of MC for the pre-defined native biopsy indications is shown in Table 4. The three most common indications were ‘acute kidney injury (AKI) query cause’, ‘chronically reduced estimated glomerular filtration rate (eGFR) with proteinuria’ and ‘nephrotic syndrome’ respectively. The highest incidence of MC was in native biopsies performed for ‘AKI query cause’.

**Table 4:** Distribution and incidence of MC for the pre-defined native biopsy indications.

	No. of biopsies	No. of MC	%
AKI query cause	1933	63	3.3
Chronically reduced eGFR with proteinuria	1408	30	2.1
Nephrotic syndrome	1203	15	1.2
Proteinuria with normal eGFR	721	11	1.5
Chronically reduced eGFR without proteinuria	351	4	1.1
Not specified	213	2	0.9
Assess disease activity and or response to treatment	149	0	0.0
Other (specify)	99	2	2.0
Normal eGFR and non-visible haematuria without proteinuria	90	0	0.0

The distribution and incidence of major complications for the pre-defined transplant biopsy indications is shown in Table 5. The three most common indications for transplant biopsy were ‘AKI query cause’, ‘chronically deteriorating transplant function without proteinuria’ and ‘achieved transplant function lower than expected’. The highest incidence of MC was in transplant biopsies performed for the indication ‘preserved transplant function and proteinuria’ (4.9%), although only 41 biopsies were performed for this indication.

**Table 5:** Distribution and incidence of MC for the pre-defined transplant biopsy indications.

	No. of biopsies	No. of MC	%
AKI query cause	856	4	0.5
Chronically deteriorating transplant function without proteinuria	397	6	1.5
Achieved transplant function lower than expected	269	3	1.1
Chronically deteriorating transplant function and proteinuria	238	4	1.7
Surveillance during delayed graft function	206	4	1.9
Assess disease activity and or response to treatment	147	3	2.0
Other (specify)	94	1	1.1
Protocol (surveillance) biopsy	45	0	0.0
Preserved transplant function and proteinuria	41	2	4.9
Features of pancreas rejection in SPK	8	0	0.0
Not specified	8	1	12.5

SPK, simultaneous pancreas and kidney transplant.

### Serum creatinine

Serum creatinine result at the time of biopsy was reported for 97.0% of native biopsies and 99.2% of transplant biopsies. The distribution and incidence of MC by quartile of serum creatinine at the time of biopsy is shown in Table 6. For native biopsies there was a significant association between increasing serum creatinine at the time of biopsy and increasing incidence of MC with 3.2% MC in the highest quartile (serum creatinine >271 µmol/L) reducing in each quartile to 1.3% in the lowest quartile (serum creatinine <166 µmol/L) (*P *= .002). The trend remained when biopsies for the indication AKI or unknown were excluded but was no longer statistically significant. There was no obvious association between quartiles of serum creatinine at the time of biopsy and incidence of MC in transplant biopsies irrespective of inclusion of AKI as the indication.

**Table 6:** Distribution and incidence of MC by quartile of serum creatinine at the time of biopsy.

	All				Excluding indication AKI			
	No. of biopsies	No. of MC	% MC	*P*	No. of biopsies	No. of MC	% MC	*P*
Native biopsy								
sCr <99 µmol/L	1499	20	1.3	<.002	1399	16	1.1	.37
sCr 99–165 µmol/L	1449	26	1.8		1113	19	1.7	
sCr 166–271 µmol/L	1467	32	2.1		895	17	1.9	
sCr >271 µmol/L	1442	48	3.2		422	9	2.1	
Not reported	184	1	0.5					
Transplant biopsy								
sCr <159 µmol/L	574	5	0.9	.28	342	4	1.2	.67
sCr 159–214 µmol/L	554	11	1.9		350	8	2.2	
sCr 215–358 µmol/L	563	6	1.1		352	6	1.7	
sCr >358 µmol/L	564	5	0.9		375	5	1.3	
Not reported	131	1	0.8					

### Biopsy diagnosis

The native kidney biopsy diagnosis was the ERA Primary Renal Diagnosis code that was felt by the reporting nephrologist to explain best the indication for that biopsy. Ninety-three coded diagnostic terms were used by the reporting nephrologists for the native biopsies. It is worth noting that a small number of these were repeat biopsies with the same diagnosis in individual patients over the 10-year period.

The incidence of MC in biopsies in each of these diagnosis categories is shown in Supplementary material 2. For diagnoses with >100 cases the four individual diagnoses with the highest incidence of major complications were chronic kidney disease/chronic renal failure (CKD/CRF) aetiology uncertain (4.7%), ischaemic nephropathy/microvascular disease (3.8%), insufficient renal tissue for diagnosis (3.4%) and systemic vasculitis—ANCA negative (3.1%).

The distribution and incidence of MC for the reported transplant biopsy diagnosis using the pre-defined Scottish Renal Registry transplant biopsy diagnosis coded terms is shown in Supplementary material 2. Of the transplant diagnostic categories with >50 cases, the highest incidence of MC were ‘insufficient renal tissue for diagnosis’ (7.1%), ‘calcineurin inhibitor toxicity’ (6.5%) and ‘donor disease’ (2.5%).

### Time since transplant

**Figure 2:**
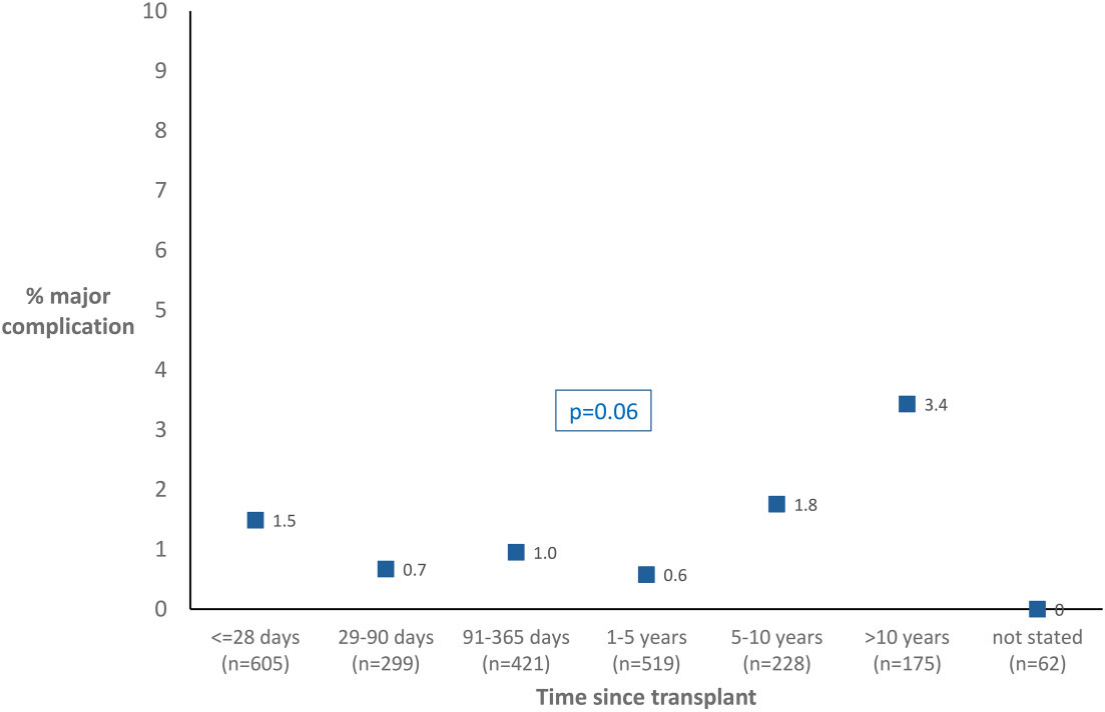
Time since transplant and the incidence of MC of transplant biopsy.

For biopsies of transplant kidneys, time since last transplant was categorized in to meaningful time periods and the distribution of these and associated incidence of MC is shown in Fig. 2. The majority of transplant biopsies were done in the first year after transplant, but the highest incidence of MC was in biopsies done >10 years after transplant (3.4%).

### Deaths

The mean age at time of biopsy of the nine patients that experienced death attributed to the native biopsy was 64.1 years (range 30.8–79.8). The indications for biopsy in these cases were: nephrotic syndrome (*n* = 5), AKI (*n* = 2), chronically reduced eGFR with proteinuria (*n* = 1) and chronically reduced transplant function (*n* = 1). The median time from biopsy to death was 9 days (range 1–30). The single death attributed to transplant biopsy occurred 13 days after biopsy.

## DISCUSSION

To our knowledge this is the first report of a complete national analysis of verifiably attributed MC following both native and transplant kidney biopsy over an extended period. The overall MC incidence for native kidney biopsy of 2.1% is similar to a recent meta-analysis of complications of native kidney biopsy including 87 studies and 118 000 biopsies performed between 1983 and 2018 [1]. It is lower, however, than 5% reported both in a recently published Italian multicentre study of 5304 biopsies from 54 centres over an 8-year period [8] and a nationwide French study of 52 138 native biopsies from 2010 to 2018 [9]. This might reflect differences in definition of MC. In our study blood transfusion was regarded as a MC only if it was felt, by the reporting nephrologist to be a direct consequence of bleeding after the kidney biopsy whereas any blood transfusion within 8 days of biopsy, identified through linkage, was regarded as a MC of biopsy in the French study. The Italian study included haematoma >5 cm post-biopsy or fall in haemoglobin concentration of >20 g/L as a MC even if these did not lead to intervention which may explain the difference compared with our study.

We found a lower incidence of MC in transplant biopsies of 1.2%. This is also lower than 3.18% in a meta-analysis of 72 published studies that included 40 082 transplant biopsies from 2000 to 2020, although in that analysis gross haematuria was included as a MC and there was acknowledged wide heterogeneity in definitions and healthcare setting. It is also lower than 4.6% reported in a French national study of 28 034 transplant biopsies performed between 2008 and 2019 [10]. We speculate the lower incidence of MC in transplant biopsies is due to the transplant kidney usually being easier to reach with the biopsy needle than the native kidney.

By including other information from the time of biopsy including coded indication, serum creatinine and diagnosis, we were able to explore other associations that have been suggested by previous studies. We found a U-shaped relationship between age and incidence of MC of native kidney biopsy, although it should be emphasized that the number of biopsies at the extremes of age within our study was relatively small. This contrasts with the French national study that identified increasing age as a risk factor for MC [9] but again this might be because all blood transfusions within 8 days of biopsy were regarded as a MC in the French study and older patients are more likely to have other reasons for needing blood transfusion in the days after kidney biopsy that are not necessarily a consequence of bleeding after kidney biopsy.

MC of both native and transplant biopsy were more frequent in women than men. This is consistent with some previous studies [9, 11]. It is not clear why MC of biopsy should be more common in women than men but we speculate that in native biopsies it may relate to relatively deeper penetration of the biopsy needle in to the smaller female kidney and differences in tissue composition in the perinephric area, meaning that bleeding is more contained in men.

We found identical incidence of MC for native and transplant biopsies performed by nephrologists and radiologists which has important implications in the ongoing debate about whether nephrologists should continue performing biopsy procedures [12].

The analysis of MC by volume of procedures carried out by centres was reassuring despite a wide range, with the smallest centre performing 150 native biopsies during the study period compared with 1848 in the largest centre.

We found that the incidence of MC was very similar in centres that continue aspirin compared with centres that stop aspirin for non-urgent biopsies. This is consistent with evidence from one previous study that was able to compare directly biopsies done in individual patients who had stopped or continued aspirin [13] and with the guideline for kidney biopsy of the Australia and New Zealand Society of Nephrology [14].

Our analysis of indication for native biopsy indicates that the risk of MC is highest in patients undergoing biopsy for investigation of unexplained AKI which is consistent with previous reports [1, 10, 11]. However the incidence of MC was not higher in transplant biopsies performed for the same indication where no indication was associated with an obvious high risk of MC.

Some previous reports have suggested that worsening kidney function is associated with increasing risk of MC and hypothesized that this is due to reduced platelet function in uraemia [11]. However, there is potential for confounding as these are also likely to be the patients undergoing biopsy for investigation of native AKI. We explored this and confirmed that patients with worsening kidney function had increasing risk of MC. When we re-analysed after excluding biopsies done for the indication AKI the association with serum creatinine diminished. AKI as an indication for transplant biopsy was not associated with a higher risk of MC. We speculate that subjects with native AKI are often systemically unwell and have quite advanced AKI by the time they reach kidney biopsy making biopsy more challenging and the patient less resilient to withstand bleeding complications. In contrast transplant AKI is likely to be identified earlier due to close monitoring of kidney function and the common causes of biopsied transplant AKI do not usually make the patient systemically unwell.

The analysis of native biopsy diagnosis and risk of MC is challenging because there are so many different diagnostic possibilities meaning that many diagnoses were made in only a relatively small number of patients. It is noteworthy that the diagnoses with a relatively high incidence of MC were ‘glomerulonephritis—histologically indeterminate’, ‘CKD/CRF aetiology uncertain’ and ‘insufficient renal tissue for diagnosis’; it may be that difficulties in getting enough tissue for a diagnosis were the reason for both the complication and the allocation of these diagnoses. It would be interesting to analyse any association between number of needle passes and the risk of MC in this context but unfortunately number of needle passes is not part of the core data set of the biopsy registry. One previous study suggested that renal amyloid was a risk factor for major bleeding complications [15] but we found three MC in 165 biopsies with a diagnosis of amyloid (1.8%), similar to the incidence of the overall cohort. Our data do not support any particular histological diagnosis being a definite increased risk for MC.

Categorizing transplant biopsy diagnosis is even more challenging than for native biopsies. Again, ‘insufficient tissue for diagnosis’ was associated with a high risk of MC.

Time since transplant appeared to be associated with increased risk of MC, particularly >10 years. It is well recognized that old transplant kidneys often have a thick capsule and can be difficult to biopsy.

Death is the most important complication. This was fortunately rare but occurred as a direct consequence of 0.15% of native biopsies and in one transplant biopsy. This compares with incidence of death attributable to native biopsy of 0.06% in a systematic review of complications of native kidney biopsy [1].

It is noteworthy that there were no nephrectomies as a result of native kidney biopsy. This provides further evidence to reassure nephrologists who regard a single functioning kidney as a contraindication to native biopsy although it should be acknowledged that embolization of a branch of the renal arterial supply to stop bleeding could result in loss of a significant portion of functioning kidney. In addition, it is possible that nephrectomy was considered but not attempted in some of the small number of deaths attributed to biopsy.

Our analysis has several strengths. It is a complete dataset of all native and transplant kidney biopsies performed in all nephrology centres in one national health service which minimizes the risk of ascertainment bias. Since the biopsy registry was created in 2005 the steering group has focussed on simplifying data collection strategies to ensure all centres can achieve near 100% coverage. It is possible that occasional procedures were not reported but we are confident the number of these will be very low. All biopsies in the nine centres are performed in a standard way using similar techniques. There are minor variations in preparation between centres such as whether or not to stop aspirin and who performs the biopsy and we were able to explore the association of these with major complications. We defined codes for MC in advance of data collection which increases confidence in the analysis. Similarly, we use pre-defined terms for indication and diagnosis. We were able to compare MC of native and transplant biopsies in the same study.

There are also limitations that should be acknowledged. There is always a concern about under-reporting of MC particularly in multicentre registry analyses and this cannot be excluded without direct verification of individual records which was beyond the scope of this study. However, as annual reporting and discussion of MC at a national level is a key feature of the registry, we believe there is a culture of openness within the registry that encourages reporting and our reported MC incidence is similar to other published series. We did not have information on some other factors that might be relevant to the incidence of MC that have been reported in previous studies such as needle gauge, number of passes, liver disease, blood pressure, haemoglobin level, routine post-biopsy ultrasound scan and recent plasmapheresis. Limited granularity is an inevitable consequence of a registry such as the Scottish Renal Registry where the desire for 100% reporting of all subjects can only be achieved by restricting the minimal required dataset to focus on important and available variables. The analysis comparing centres that stop aspirin in advance of elective biopsies and those that do not was limited because we did not collect data on stopping aspirin at an individual patient level.

## CONCLUSION

MC of kidney biopsy in the modern era are rare but are more common with native than transplant biopsy, more common in women than men, and include death. The rarity of major complications mean it is important that there is ongoing multicentre prospective reporting of all kidney biopsies and MC so that patients and clinicians have the most precise information when discussing risk at the time of proposed kidney biopsy.

## Supplementary Material

sfaf196_Supplemental_Files

## Data Availability

The permission to collect and store these data within Public Health Scotland did not include permission to share the data. Separate ethical approval would be required to make these data available to external parties.
