# Supplementary material for: Major complications of percutaneous native and transplant kidney biopsy: a complete 10-year national prospective cohort study
Source: Clin Kidney J. 2025 Jun 23;18(7):sfaf196. doi: 10.1093/ckj/sfaf196 (PMC12259278; doi:10.1093/ckj/sfaf196)
Supplement: sfaf196_Supplemental_Files [file sfaf196_supplemental_files.zip › 512 Supplementary material 1.docx]

**Supplementary material 1**

Distribution and incidence of major complications in all 92 native renal biopsy coded diagnostic terms used by the reporting nephrologists.

|  | **number of biopsies** | **number of major complications** | **%** |
| --- | --- | --- | --- |
| **IgA nephropathy** | 817 | 19 | 2.3 |
| **Tubulointerstitial nephritis** | 527 | 7 | 1.3 |
| **Membranous nephropathy - idiopathic** | 432 | 3 | 0.7 |
| **Microscopic polyangiitis** | 389 | 6 | 1.5 |
| **Minimal change nephropathy** | 296 | 1 | 0.3 |
| **Systemic lupus erythematosus / nephritis** | 295 | 6 | 2.0 |
| **Primary focal segmental glomerulosclerosis (FSGS)** | 291 | 7 | 2.4 |
| **Diabetic nephropathy in type II diabetes** | 247 | 2 | 0.8 |
| **Granulomatosis with polyangiitis** | 226 | 4 | 1.8 |
| **Chronic kidney disease (CKD / CRF) aetiology uncertain** | 172 | 8 | 4.7 |
| **acute tubular injury** | 153 | 3 | 2.0 |
| **Chronic hypertensive nephropathy** | 150 | 2 | 1.3 |
| **Other (specify)** | 131 | 7 | 5.3 |
| **Ischaemic nephropathy / microvascular disease** | 130 | 5 | 3.8 |
| **Systemic vasculitis - ANCA negative** | 129 | 4 | 3.1 |
| **Insufficient histological evidence from kidney biopsy for diagnosis** | 128 | 1 | 0.8 |
| **not stated** | 110 | 0 | 0.0 |
| **AL amyloid secondary to plasma cell dyscrasia** | 109 | 1 | 0.9 |
| **Glomerulonephritis** | 86 | 5 | 5.8 |
| **Insufficient renal tissue for diagnosis** | 83 | 3 | 3.6 |
| **Focal and segmental proliferative glomerulonephritis** | 75 | 0 | 0.0 |
| **Mesangial proliferative glomerulonephritis** | 73 | 1 | 1.4 |
| **Mesangiocapillary glomerulonephritis type 1** | 70 | 5 | 7.1 |
| **No significant abnormality** | 66 | 1 | 1.5 |
| **Drug-induced tubulointerstitial nephritis** | 65 | 1 | 1.5 |
| **Henoch-Schonlein purpura / nephritis** | 60 | 1 | 1.7 |
| **Diabetic nephropathy in type I diabetes** | 54 | 1 | 1.9 |
| **Anti-Glomerular basement membrane disease** | 52 | 2 | 3.8 |
| **Malignant / accelerated hypertensive nephropathy** | 52 | 4 | 7.7 |
| **Myeloma cast nephropathy** | 50 | 1 | 2.0 |
| **Light chain deposition disease** | 49 | 1 | 2.0 |
| **Thin basement membrane disease** | 48 | 0 | 0.0 |
| **Immunotactoid / fibrillary nephropathy** | 37 | 2 | 5.4 |
| **No renal tissue** | 36 | 1 | 2.8 |
| **AA amyloid secondary to chronic inflammation** | 35 | 0 | 0.0 |
| **Glomerulonephritis - secondary to other systemic disease** | 34 | 2 | 5.9 |
| **Renal sarcoidosis** | 32 | 0 | 0.0 |
| **Diffuse endocapillary glomerulonephritis** | 27 | 1 | 3.7 |
| **Tubulointerstitial nephritis with uveitis (TINU)** | 27 | 0 | 0.0 |
| **Focal segmental glomerulosclerosis FSGS secondary to obesity** | 22 | 0 | 0.0 |
| **Renal amyloidosis** | 20 | 2 | 10.0 |
| **Tubulointerstitial nephritis associated with autoimmune disease** | 20 | 0 | 0.0 |
| **Complement component 3 glomerulopathy** | 19 | 0 | 0.0 |
| **Cryoglobulinaemia secondary to systemic disease** | 18 | 0 | 0.0 |
| **Membranous nephropathy - drug induced** | 15 | 0 | 0.0 |
| **Churg-Strauss syndrome** | 14 | 0 | 0.0 |
| **FSGS secondary to hyperfiltration injury** | 13 | 0 | 0.0 |
| **Idiopathic rapidly progressive (crescentic) glomerulonephritis** | 13 | 0 | 0.0 |
| **Membranous nephropathy - malignancy associated** | 13 | 2 | 15.4 |
| **Atheroembolic renal disease** | 12 | 0 | 0.0 |
| **Alport syndrome** | 11 | 0 | 0.0 |
| **Atypical haemolytic uraemic syndrome (HUS) - diarrhoea negative** | 10 | 2 | 20.0 |
| **Infiltration by lymphoma** | 10 | 0 | 0.0 |
| **Thrombotic microangiography** | 10 | 0 | 0.0 |
| **Mesangiocapillary glomerulonephritis type 2 (dense deposit disease)** | 8 | 0 | 0.0 |
| **Mesangiocapillary glomerulonephritis type 3** | 8 | 0 | 0.0 |
| **Acute pyelonephritis** | 7 | 0 | 0.0 |
| **Nephropathy due to ciclosporin** | 7 | 2 | 28.6 |
| **Tubulointerstitial nephritis with granulomata** | 7 | 0 | 0.0 |
| **Chronic urate nephropathy** | 5 | 0 | 0.0 |
| **Nephropathy due to lithium** | 5 | 0 | 0.0 |
| **Fabry disease** | 4 | 0 | 0.0 |
| **IgA nephropathy secondary to liver cirrhosis** | 4 | 0 | 0.0 |
| **Nephropathy due to tacrolimus** | 4 | 1 | 25.0 |
| **Essential mixed cryoglobulinaemia** | 3 | 0 | 0.0 |
| **Membranous nephropathy - infection associated** | 3 | 0 | 0.0 |
| **Nephropathy related to HIV** | 3 | 0 | 0.0 |
| **Renal scleroderma / systemic sclerosis** | 3 | 0 | 0.0 |
| **Cryoglobulinaemia secondary to hepatitis C** | 2 | 0 | 0.0 |
| **Familial interstitial nephropathy** | 2 | 0 | 0.0 |
| **Focal segmental glomerulosclerosis (FSGS) secondary to HIV** | 2 | 0 | 0.0 |
| **Glomerulopathy with giant fibrillar deposits** | 2 | 0 | 0.0 |
| **Hypercalcaemic nephropathy** | 2 | 0 | 0.0 |
| **Nephropathy due to analgesic drugs** | 2 | 0 | 0.0 |
| **Nephropathy due to pre-eclampsia** | 2 | 0 | 0.0 |
| **Primary hyperoxaluria** | 2 | 0 | 0.0 |
| **Sickle cell nephropathy** | 2 | 0 | 0.0 |
| **Acute kidney injury due to rhabdomyolysis** | 1 | 0 | 0.0 |
| **Acute urate nephropathy** | 1 | 0 | 0.0 |
| **Calcium oxalate urolithiasis** | 1 | 0 | 0.0 |
| **Calculus nephropathy / urolithiasis** | 1 | 0 | 0.0 |
| **Enteric hyperoxaluria** | 1 | 0 | 0.0 |
| **Familial amyloid secondary to protein mutations** | 1 | 0 | 0.0 |
| **Familial focal segmental glomerulosclerosis FSGS autosomal dominant** | 1 | 0 | 0.0 |
| **Focal segmental glomerulosclerosis FSGS secondary to previous vasculitis** | 1 | 0 | 0.0 |
| **IgM - associated nephropathy** | 1 | 0 | 0.0 |
| **Kidney tumour** | 1 | 0 | 0.0 |
| **Medullary cystic kidney disease type II** | 1 | 0 | 0.0 |
| **Nephropathy due to cisplatin** | 1 | 0 | 0.0 |
| **Radiation nephritis** | 1 | 0 | 0.0 |
| **Thrombotic thrombocytopenic purpura (TTP)** | 1 | 0 | 0.0 |
| **Transitional cell carcinoma** | 1 | 0 | 0.0 |

**Supplementary material 2**

*Distribution and incidence of major complications for the transplant biopsy diagnostic categories.*

*TCMR = t cell mediated rejection; IFTA = interstitial fibrosis and tubular atrophy, BKVAN = Bk virus associated nephropathy, ABMR = antibody mediated rejection, iIFTA = inflammation in areas of interstitial fibrosis and tubular atrophy*

|  | **number of biopsies** | **number of major complications** | **%** |
| --- | --- | --- | --- |
| **acute tubular injury** | 363 | 6 | 1.7 |
| **Other** | 261 | 1 | 0.4 |
| **TCMR (2A, 2B or 3)** | 194 | 1 | 0.5 |
| **no significant histopathological abnormality** | 176 | 4 | 2.3 |
| **IFTA** | 160 | 0 | 0.0 |
| **TCMR borderline, 1A or 1B** | 371 | 2 | 0.5 |
| **BKVAN** | 110 | 0 | 0.0 |
| **recurrence of primary renal disease** | 122 | 2 | 1.6 |
| **donor disease** | 79 | 2 | 2.5 |
| **rejection: chronic, active ABMR** | 79 | 1 | 1.3 |
| **calcineurin inhibitor toxicity** | 62 | 4 | 6.5 |
| **rejection: chronic ABMR** | 60 | 1 | 1.7 |
| **rejection: mixed ABMR & TCMR** | 56 | 0 | 0.0 |
| **Insufficient renal tissue for diagnosis ^a^** | 56 | 4 | 7.1 |
| **rejection: acute / active ABMR** | 44 | 0 | 0.0 |
| **not stated** | 38 | 0 | 0.0 |
| **infection (other than BKVAN)** | 37 | 0 | 0.0 |
| **iIFTA** | 25 | 0 | 0.0 |
| **rejection: chronic allograft arteriopathy** | 11 | 0 | 0.0 |
| **Thrombotic microangiography** | 5 | 0 | 0.0 |

^a^ includes 9 cases with no renal tissue
